# What is (synthetic) life? basic concepts of life in synthetic biology

**DOI:** 10.1371/journal.pone.0235808

**Published:** 2020-07-28

**Authors:** Sandra Fernau, Matthias Braun, Peter Dabrock

**Affiliations:** Chair of Systematic Theology II (Ethics), Friedrich-Alexander-University Erlangen-Nürnberg, Erlangen, Germany; US Army Engineer Research and Development Center, UNITED STATES

## Abstract

One of the central aims of synthetic biology (SB) is to better understand the mechanisms of life by trying to develop and synthesize new forms and perhaps modes of life. While the question of what is life has occupied mankind for centuries, there is a lack of empirical research examining the basic concepts of life scientists within SB themselves refer to and build on. In order to gain insights into these fundamental concepts, we conducted a qualitative interview study with scientists working in the field of SB. The aim was to gain a better understanding of the underlying understandings, principles, and characteristics of (synthetic) life on the one hand, and the entangled consequences for the conducted experiments and studies as well as the pursued scientific approaches. We identified four primarily underlying basic concepts of life which serve as a fundamental framework for current and further scientific research within SB and have implications for research questions, approaches and aims as well as for the evaluation of scientific results.

## 1. Introduction

The fundamental question of what is life is and how it can be defined has occupied mankind for centuries and has recently gained increasing attention in several fields of emerging biotechnologies, especially within synthetic biology (SB) [1–3]. In general, SB is understood as an umbrella term covering a diverse field of scientific practices with different agendas and methodologies. Hence, it combines varied disciplines, such as biology, physics, chemistry, and engineering. SB promises to provide profound new insights about the characteristics, principles, and origins of life and nature [4, 5] as its underlying scientific objective is to gain a deeper and more comprehensive understanding of biological organisms, their features and central organizational principles. This is achieved by conceptualizing and finally constructing novel biological systems consisting of newly modularized components. The vision of generating new forms of life–as well as the related successes postulated by scientists within this field [6]–challenges traditional concepts of life and nature as well as classical distinctions between living and non-living matter [5, 7, 8]. Thus, these (expected) developments urge us to reconsider questions related to the central principles and characteristics of life and living and stimulate both the scientific and the ethical debate on defining life.

The question of what life is and of whether or not it is a meaningful or even possible scientific endeavor to search for a definition of life has a long-reaching history [9]. While Aristotle argued that life has to be understood as a fundamental, irreducible property of nature [10], it was Descartes (among others) who defined life as a universal mechanism [11]. During his engagement with the mechanistic definition of life, Kant responded by defining life as the capacity for self-determination [12]. Thus, in his understanding, life is a form of causality, which itself cannot simply be explained in an empirical-causal way. As a consequence, several scientists built upon this idea of Kant and merged it with Aristotle’s concept. Against the mechanical perspective, they postulated that living organisms contain some non-physical element, which fundamentally distinguishes them from non-living entities. This more or less strict dichotomy between mechanistic and vitalistic definitions of life spilled over into the twentieth century, especially during the time that biochemists were defining their field as a separate discipline from chemistry or physiology. There were several approaches in both the natural sciences as well as philosophy, trying to revive the debate again. Within the natural sciences this was the famous work of Schrödinger [13] and within philosophy the work of Whitehead [14] as well as of Plessner [15].

The current debate has revisited the concepts and theories of life. This is especially the case because natural scientists, as well as philosophers, are facing the question of whether or not it is possible to create novel forms of life from scratch [16–18]. Despite manifold attempts on a definition of life, there is no consensus on the necessary and sufficient conditions for life. This is even true for the attempt to define life in terms of a list of distinctive properties that distinguish living organisms from non-living [19, 20]. Although there is some overlap, there is a set of similar but often not congruent lists, each of them trying to comprehensively define life [21]. Additionally, these lists of criteria seem to function as fuzzy concepts by including or excluding entities, which tend to blur the lines between living and non-living [22]. This applies for instance to viruses, which are not able to reproduce on their own, or crystals which–under some circumstances–seem to be able to grow. One possible solution in dealing with these challenges and the fact that no scientific consensus can be reached may be to reject the assumed need and related attempts to establish a definition of life. This may be due to the notion that life is an irreducible fact about the ‘natural world’ and the related incapability of definitions to mark out natural kinds, or the notion that life is an irreducible, fuzzy, vague and too broad concept [23–26].

In general, there are two distinct kinds and uses of definitions of life that are discussed in the literature most predominantly [3]: Fixed definitions with an ontological claim, which aim at demarcating life by carving out natural kinds and are based on the generalization of characteristics of all forms of living [26] on the one hand and flexible “operational definitions” [3] with an instrumental claim serving as theoretical tools that guide debates and experiments [27] on the other. As Bich and Green (3) argue, the aforementioned critique addresses primarily the first category of definitions of life and assumes that a definition’s basic purpose is to establish a set of universal criteria of living objects. In contrast, they indicate that within the current scientific discourse in emerging biotechnologies, such as SB, the issue of defining life has a more pragmatic utility in the sense of operational definitions that serve as theoretical and epistemic tools in scientific practice. By means of exemplary definitions of life of leading scientists within the field of SB [e.g. 1, 27] they show that these conceptualizations influence the choice of the conducted experiments, the research programs as well as the assessment of scientific results.

While assuming, that there is a fundamental entanglement between the used fundamental concepts on life and the used approaches and pursued goals within SB [28–31], there is only little knowledge about the notions and concepts of life which are used by the scientist themselves. Against this background, this article first scrutinizes the underlying epistemological presumptions of life and living matter. Second, we ask which implications the different epistemological concept of life has on the conducted practices and endeavors within SB. In spite of the widespread assumption on the anticipated scientific advances within SB, there is a lack of empirical research examining the specific perceptions and underlying notions of life and living matter of scientists working in the field of SB. To fill in this research gap, we conducted a qualitative interview study with scientists that addresses the following main questions: 1.) How do researchers in the field of SB conceptualize (synthetic) life and living matter? 2.) Which basic principles, characteristic features, and distinguishing dimensions underlie their approaches to life?

## 2. Study design

The present interview study is based on 20 semi-structured interviews with scientific experts working as researchers in the field of SB. The research team conducting the study holds expertise in sociology, ethics and biology and has a long-standing expertise on the ethical and philosophical issues with regard to SB. This has played an important role in identifying the scope of this study and helps us to get into touch with the leading figures in the field of SB.

The general focus of our investigation is on the scientific (and not on the more application-oriented, industry-driven) part of SB. This is due to the fact that merely scientific approaches within this field directly address questions of the concept, principles and origins of life following a specific research methodology and agenda [32, 33]. The interview study was conducted from autumn 2017 until the beginning of 2018. The interviewees were selected by a contrasting sampling method in order to obtain a heterogenic sample with wide-ranging structural variation within the field of SB. Such a contrasting case selection during data collection and analysis aims at exploring the heterogeneity of the research field in order to enable a generalization of the reconstructed patterns even on the basis of a relatively small sample [34]. Initially, we predefined contrasting typological sampling criteria in terms of gender, scientific background, professional position and the research approach of the scientists. On the basis of these features, in a first step, we selected and recruited via email leading scientific experts who are internationally recognized for their work within the field of SB (h-index > 19). In a second step, utilizing snowball-sampling [35], we contacted further researchers (mainly postdocs) following the recommendations of the initially interviewed senior scientists, who thus served as multiplicators. In total, we asked 24 researchers to participate in the study. Two of the senior researchers, as well as two of the postdocs, were not able to attend because of time issues. The overall sample provided a range of experts in SB across Germany with different scientific backgrounds and professional positions (see Table 1): it consisted of senior scientists, who were all full professors (n = 11), as well as junior scientists (postdocs) in a mid-level position (n = 9) and included experts being trained as biologists and biochemists (n = 10), physicists and biophysicists (n = 5), chemists (n = 3) and engineers (n = 2).

**Table 1:** Sample structure of the interview study.

Total	
Participants	
Total	20
Female	7
Male	13
Scientific backgrounds	
Biology (including biochemistry)	10
Physics (including biophysics)	5
Chemistry	3
Engineering	2
Professional position	
Senior scientist	11
Junior scientist	9
Research approach	
Bottom-up	17
Top-down	3

The interviews were planned as semi-structured, guideline-based expert interviews [36, 37], using a set of open questions on specific pre-existing topics in a flexible running order. The underlying strategy was to account for both comparability and openness, aimed at maintaining a continuous narrative process of the respondents. The interview guide addressed four main topics: 1.) perspectives on SB and self-conception as a researcher in this area, 2.) approaches to life, 3.) assessment of current research activities and envisioned future prospects of SB, 4.) anticipated ethical and societal implications of SB. Depending on the preferred language of the respective scientist, the interviews have been conducted either in German or English. The interviews lasted between 25 minutes and almost one and a half hours, with an average duration of around 45 minutes. As usual, informed consent for participation in the interview study was obtained in writing from all respondents.

The interviews were audio-recorded and transcribed word-for-word for further analysis. In cases where the interview has been conducted in German, the transcript has been translated by a native speaker and been double-checked by the research team [38]. All personal identifying data of the participants were anonymized. The central aim of the analysis was to go beyond the manifest content of the respondents’ statements by revealing underlying patterns of interpretation and argumentation as rather implicit structures of meaning. For this purpose, we conducted theoretical coding according to central principles of the approach of Grounded Theory [39–41]. The overarching categories and their assigned codes were mainly developed inductively, which means that they are based on findings from the empirical material. Furthermore, the development of the semi-structured interview guide was embedded into an in-depth analysis of the ethical and philosophical literature regarding concepts of (synthetic) life. The reason for this was to develop and proof the interview design against the background of the current state of debate. As a first step, a sequential single-case analysis was carried out by means of open coding in order to reconstruct the different perceptions and conceptualizations of life in detail and to build initial codes. In a second step, following axial coding, the relations between the different cases and codes were explored in-depth, and the most relevant codes were grouped around a few central categories with certain subcategories. All categories and codes were validated internally by an intercoder consensus of three researchers who discussed their interpretative results until a shared understanding was reached. On the basis of minimal and maximal contrast, we identified four basic concepts of life, which all interviewed scientific experts could be assigned to. The crucial subcategories for conceptual differentiation with regard to the underlying patterns of argumentation addressed the following relations, understood here as distinguishing dimensions of life as a border phenomenon: 1.) differentiation between living and non-living, 2.) differentiation between natural/non-synthetic and artificial/synthetic, especially with regard to the assessment of the generation of synthetic organisms, 3.) differentiation between life and living objects.

## 3. Basic concepts of life in synthetic biology

Based on the empirical data we reconstructed four basic concepts of life, which can be understood as the interviewees’ experience-based approaches to (synthetic) life. As heuristics they do not operate with predefined, specific criteria that are put into a “set of mutually dependent necessary conditions” [3] which is a crucial aspect of (operational) definitions. Nevertheless, they go beyond the simple collection of “tentative criteria” [42] for life because of their overarching, more abstract, and generalizing character and claims. Hence, the present basic concepts of life can be located at a preliminary stage of operational definitions of life mentioned at the beginning of the paper. Although these concepts are more fundamental and less concrete than definitions, they play an important role in the respective scientific practices.

The reconstructed basic concepts of life are: 1.) life as a stage model based on a list of properties of living matter, 2.) life as a non-definable continuous process starting with the origin of life, 3.) life as a hitherto undiscovered general principle, and 4.) life as a relational, intuitive concept depending on subjective views. In the following sections each conceptualization will first be described in a general manner, and second in terms of the three distinguishing dimensions underlying the patterns of argumentation of the interviewees. To provide a better overview, Table 2 shows all the results in advance.

**Table 2:** Overview of the concepts of life and their underlying distinguishing dimensions.

	Differentiation between living and non-living	Differentiation between natural/non-synthetic and artificial/synthetic	Differentiation between life and living objects
**Life as a stage model based on a list of properties of living matter**	**+/-** Difficult but possible or not clearly possible to draw a line by defining different stages and describing gradual transitions	**+/-** Synthetic objects resort to natural processes and also consist of synthetic components: mix of imitating nature and constructing from scratch	**+/-** Differentiated only on an implicit level: the criteria address properties of living objects as specific empirical modes of existing but do not describe life itself as an abstract concept
**Life as a non-definable, continuous process starting with the origin of life**	**-** Not possible to draw a line due to fluent transitions and processuality of life	**-** Synthetic objects resort to natural processes as only single components can be constructed synthetically: imitating and manipulating nature	**-** Not differentiated as life and living objects are dynamic and open-ended
**Life as a hitherto undiscovered general principle**	**+** Possible to draw a line in future by setting an abstract distinction based on a switch mechanism after identifying the generic principle of life	**+** Basic principles of natural and synthetic objects are similar: envisioned constructing from scratch	**+** Hierarchically differentiated: living addresses specific organic forms of existence, life addresses an overarching, structuring principle, which all living objects are based on
**Life as a relational, intuitive concept depending on subjective views**	**+/-** Objectively not possible to draw a line as any distinction relies on subjective ascriptions	**+/-** Not clearly definable to what extent objects are non-synthetic or synthetic: mix of imitating nature and constructing from scratch, with both based upon subjective judgements	**+** Abstractly differentiated: living addresses specific organic forms of existence definable based on ascriptions, life addresses a universal principle of all possible living entities and cannot be defined

### 3.1 Life as a stage model based on a list of properties of living matter

Within this conception, life is described as several properties, which are shared by all forms of living matter. Hence, this conception reflects the understanding of life that underpins traditional biology [20, 21, 43–45]: it is based on a set or list of biological and physical properties characterizing living organisms. These properties can be understood as fundamental features of living matter. When viewed together, they serve to define life. Almost half of the interviewees could be assigned to this conception, which is thus the most prevalent approach to defining life in our sample. The properties of living organisms that this group of interviewees mentioned frequently (in this wording or similar) were: non-equilibrium, energy supply, information transfer, autopoiesis, metabolism, growth and development, reproduction or division, motility, and interaction with the environment. This list suggests that the different properties are closely related and complement each other. Depending on how many properties are met by a living object a distinction can be drawn between different levels of organization of living matter. Therefore, life can be understood as a stage model or a “scale” (interviewee 20), in which living objects meet more or less of these properties. Consequently, there are “graduated forms of life, from very complex to more basic forms of life” (interviewee 9), which means that there are higher and more primitive organized and structured living organisms concerning their respective properties and their mutual interactions.

However, although the mentioned fundamental properties refer to traditional biological concepts of life, the interviewed synthetic biologists do not conceive of them as a given set of features, but rather as individual starting points and initial tools for constructing novel forms of living matter. Existing biological organisms have to be explored further using/utilizing SB and could, in turn, provide deeper insights about life itself. The following quote illustrates the assumed necessity for an extension of the established biological definition of life based on a list of properties:

This means that a quantification of these properties of life as well as a list of criteria should be possible indeed. It will be possible, but currently, our list still seems to be incomplete. […] Thus, I believe that there still are certain features we do not yet have identified. Consequently, the process to find a definition of life, is not yet completed as well. […] It [the definition] must be extended since within the research on biological systems, also with such synthetic approaches, other properties of life will be discovered, and possibly even a quite different classification, too. (interviewee 9)

As the cited statement reveals, within this concept it is basically feasible to define life based on a list of properties in the sense of criteria shared by living objects. Nevertheless, the interviewee stresses the need for further research on existing forms of living matter using SB to complete the existing list of properties and to probably develop a novel classification of living objects. In summary, the interviewed scientists representing this heuristic approach to life generally consider life a definable phenomenon, which can be described as a stage model based on a list–albeit incomplete and thus to be further investigated–of several properties of living organisms, emphasizing the wide-ranging contributions of SB to principles and features of life.

#### Distinguishing dimension 1: Living versus non-living

Concerning the first distinguishing relation underlying the respondents’ patterns of argumentation, this conceptualization of life includes varying assumptions on whether or not it is feasible to unambiguously distinguish between living and non-living entities. Some interviewees argue that, despite certain difficulties, in principle it is possible to draw a line between living and non-living by means of defining different stages of both modes of existence. Analogous to the above-mentioned distinction between higher and more primitive organisms, they highlight gradual transitions between living and non-living objects depending on how many properties they meet:

Perhaps we could speak of transitions [between living and non-living] by saying, yes, one has something that meets some of these properties, but not all of them. (interviewee 5)

Regarding the (envisioned) modelling of synthetic forms of living matter, this means a successive approximation to life:

Life is a combination of properties within the modules I am building since the modules do not combine all the properties. But it is one part that constitutes a living process. So, we have metabolism, I could largely say I have a metabolic network and to an extent it is, let us say, twenty percent of life. (interviewee 6)

As the quotes illustrate, these scientific experts assume that we can distinguish between living and non-living entities by defining different stages and describing gradual transitions based on the number of the respective properties natural as well as synthetically constructed organisms meet. In contrast to this, some other interviewees grouped under this conception pointed out difficulties in drawing a clear distinction between living and non-living objects, especially when it comes to constructing synthetic forms of living matter:

The question is whether there is such a boundary [between living and non-living] at all or whether we do not have to talk about a gradual transition from relational characteristics. The living systems show very complex behavioral characteristics. If I now build a synthetic system, then one usually tries to switch off certain behaviors. […] Would it suddenly no longer be a living organism because it can’t do this now? After all, it still has other characteristics that are peculiar to the natural reference system. (interviewee 9)

Despite the already cited interviewees’ suggestion that life is definable through a list of properties, at this point, with regard to synthetic organisms, the interviewee questions whether those properties are categorically met by any entity that is called alive. This is the case since an objective in constructing synthetic objects can be to simplify forms or modes of life by eliminating single biological features, which is why artificial forms of living matter do not necessarily meet all properties shared by natural forms of living matter. As a consequence, some respondents consider fluent transitions between living and non-living entities, which implies that it is not clearly possible to draw a line between both modes of existence.

#### Distinguishing dimension 2: Natural/non-synthetic versus artificial/synthetic

Within this conception of life, the generation of synthetic forms of living matter is perceived as basically feasible in future scientific practice, although the respondents assume that SB will still take natural processes and mechanisms as a paradigm in the development of novel organisms. As a consequence, there is tension regarding the assessment of current and envisioned research activities within SB: the respondents’ statements oscillate between classifying them as imitating or manipulating nature and constructing, in the sense of modelling, new artificial forms of life. The excerpt below describes the relation between SB and evolution as well as the resulting assignment of synthetic organisms:

What we are doing is the next step of evolution. Basically, we are trying to depict life in another manner, but obviously we ourselves are life. Thus, we are part of this process in this respect. […] These are synthetic systems, but I would say they have a natural character. (interviewee 6)

This quote demonstrates the notion of applying directed and thus optimized evolutionary processes as a designing aid, but nevertheless shows that the interviewee considers their own status, as well as the generated synthetic objects, as still being part of nature. Therefore, on the one hand, synthetic biologists resort to features and processes known from traditional biology and thus to natural functions of life in their research activities. In this sense, they are imitating and manipulating living objects already existing in nature, even if they are trying to simplify forms or modes of life or to make (evolutionary) processes more efficient. On the other hand, synthetic biologists use non-natural components as tools aimed at modelling new forms of synthetic organisms and thereby go beyond existing forms and modes of existence. The following quote illustrates this perspective:

We want to have something that is self-replicating, self-evolving, able to mutate, that has a compartment, is able to transfer information […], but we make ourselves completely free from what we know, and we take other building blocks, take other molecules, consider it a kit, and eventually forget what is already there, and we really start constructing from scratch so to speak. (interviewee 15)

To describe their understanding of life the interviewee uses the metaphor “kit”, which implies an understanding of life as a kind of toolbox that enables researchers to compose artificial organisms consisting of synthetically constructed “building blocks” generated from scratch. Nevertheless, the mentioned list of biological properties of living matter shows that the construction of synthetic life will still be oriented towards processes and features known from already existing forms of living matter. Accordingly, it can be assumed that within this conception of life SB is understood as a mix of imitating nature and constructing from scratch.

#### Distinguishing dimension 3: Life versus living objects

As the cited excerpts indicate, it can be stated that the interviewees do not differentiate between life and living objects on an explicit, but rather on an implicit level. In most of the narratives, various forms of labeling the different of modes of existence can be outlined: the terms “(synthetic) life” or “forms of (synthetic) life” alternate with expressions such as “living systems/organisms” or “living blocks/components/modules” and were widely used without being further questioned. As the aforementioned biological and physical criteria serve as parameters to define the fundamental features met by entities that are called alive, these criteria merely address properties of living objects understood as specific empirical modes of existing. They cannot, however, describe life as an abstract concept. Accordingly, this functional concept of life based on a list of properties can be considered an attempt to define living beings and the respective processes and mechanisms, however, it does not provide a description of life itself.

### 3.2 Life as a non-definable, continuous process starting with the origin of life

Interviewees assigned to this conception understand life as a continuous process. They focused particularly on the origins and further development of life and living matter, highlighting the processual nature of all forms of life as well as the resulting fluent transitions between living and non-living objects. Following this conceptualization, a valid, intersubjectively accepted definition of life cannot be provided by scientists as life is considered a dynamic formation without a definable starting point. As a consequence, a generalization of characteristics shared by all forms of living matter is not feasible. This point of view refers to the ongoing theoretical and epistemological discussion of whether or not it is a meaningful or even possible scientific endeavor to try to define life. Similar to contemporary suggestions [23, 24, 26] the respondents grouped under this conception emphasize that no scientific consensus on the necessary and sufficient conditions for life or living matter can be reached since life is an irreducible fuzzy concept due to its processuality and continuousness. The excerpt below demonstrates this understanding of life as an open-ended continuum implying fluent transitions between living and non-living matter:

And probably it is rather the case that we find a continuum of entities within nature, a continuum between non-living and living matter. We ourselves as scientists cannot say, okay, life starts from a virus or from sperm, what level of complexity do we need, what functions does it take? A sperm can move but it cannot divide itself. Is it alive? We cannot really say. There is a continuum and it is difficult to draw a line. At the same time, through the approach of synthetic biology we are getting into trouble, too. Basically, we are trying to build living systems, first taking only a single compartment, a vesicle, a container […] which is non-living. Then we add one building block after the other, add a function, succeed at making the thing generate energy, exhibit basic metabolisms, maybe it even divides itself, it grows, it replicates itself, and then we obviously also have to face the question, how many building blocks, how many proteins, how many functions does it take for it to correspond to what we commonly refer to as a living cell? (interviewee 18)

As the cited statement reveals, the interviewee refers to the aforementioned biological properties or features shared by living matter, which are essential for the first analyzed concept of life. However, within this conception, this–or even an extended–list of properties is not considered to be sufficient for the identification or definition of life since it does not address the issue of when life begins in terms of determining how many features have to be met to call an entity alive. In general, we can therefore conclude that within this conception it is not possible to define life either by formulating fundamental properties or by any other approach due to the fact that it has to be understood as a continuous process implying an open-ended formation. In line with this, an expected central finding of future research in the field of SB, especially concerning the investigation of transitions between non-living and living, might be that life is a non-definable phenomenon:

And I think that through our work in synthetic biology, in the long run we will realize that there is no sharp boundary between animate and inanimate matter, the whole thing is a continuum. And if you look at this continuum, perhaps from a scientific perspective it no longer makes sense to speak of this closed category of life, because we cannot close this category. (interviewee 18)

This statement demonstrates the notion that forthcoming research in SB will provide profound new insights, particularly about the origins of life, and indicates that these findings will (probably) lead to the conclusion that it is not a meaningful scientific endeavor to search for a definition of life, since life is an irreducible fuzzy concept.

#### Distinguishing dimension 1: Living versus non-living

According to the remarks mentioned above, this conceptualization of life suggests that a scientific distinction between living and non-living cannot be drawn since there are fluent transitions between both modes of existence. Again, this applies especially to the assumed origins of life, as another interviewee assigned to this conception states: “the historical development from inorganic forms to a living cell must have been a continuum” (interviewee 16). Therefore, regarding prospective findings on the origins of life as well as existing non-classifiable entities, as shown in the prominent example of viruses mentioned in the quote above, the respondents stress that assigning entities to the categories living and non-living is a problematic issue. This is due to the fact that it is not possible to define distinguishable stages of both classifications via objective parameters because the lines between living and non-living objects are considered to be blurred and cannot be kept apart.

#### Distinguishing dimension 2: Natural/non-synthetic versus artificial/synthetic

With regard to the assessed relations between naturalness and artificiality within synthetic approaches to generating life, the interviewed experts argued that despite the novelty of some proceedings, synthetic biologists still resort to natural structures and functions in their research activities and are primarily modifying and combining them in new ways by technical means. Accordingly, the respondents underlined that they cannot construct new forms of synthetic life from scratch but are merely copying pre-existing forms and building single components of living objects:

Yes, so, the aim is really just to synthetically imitate single parts, which are assigned to life. So, actually we are pursuing more of a biomimetic synthesis approach in order to be able to build cell-like structures. […] Building synthetic life really from scratch is not possible from the present point of view, it cannot be produced. (interviewee 4)

Following this evaluation, the research activities within SB can be classified as imitating and manipulating nature, implying that it is not possible to construct novel forms of life which could be labelled synthetic organisms. This assessment affects the aims pursued by SB: within this conception, the objective of research in this field is not to generate life itself but merely to reproduce single life-like features and processes of existing living objects synthetically. In line with this, the already quoted interviewee argues:

I've never had the feeling that we could in any way get close to life if we really wanted to link synthetic biology with life. […] As a chemist you really start from the molecules, and with that approach you would never be able to synthesize life. (interviewee 4)

These remarks illustrate the important role of the respective underlying conceptual approaches to life play in fields where research is guided. As shown here, they serve as a fundamental framework for current and further research within SB by shaping visions of general objectives and presumed possibilities of constructing novel forms of synthetic life, and are thereby also linked to the evaluation of potential scientific results. Referring to this basic concept of life, this means that the generation of synthetic forms of living matter is perceived as not feasible in current as well as future scientific practice and thus the scientific outcomes cannot be classified as synthetic life.

#### Distinguishing dimension 3: Life versus living objects

Regarding this conception of life, the underlying patterns of argumentation indicate that the respondents do not differentiate between life and living objects either on an explicit or an implicit level. Beyond that, as life and living are both considered dynamic and open-ended formations being part of a holistic, continuous process it is not even possible to address differences between these dimensions by means of defining distinct meanings. In line with this, the interviewees assigned to this conception used expressions such as “(synthetic) life” or “forms of (synthetic) life” and “living systems” or “living matter” synonymously in their narratives to label certain living objects as well as to describe life in general. Thus, it can be concluded that the distinction between living as a specific empirical mode of existing and life as an abstract concept is a conceptual gap of this heuristic approach.

### 3.3 Life as a hitherto undiscovered general principle

Within this conception, life is described as a hitherto undiscovered general principle. The underlying assumption is that life follows a yet unidentified comprehensive connection that goes beyond the familiar biological and physical characteristic features of forms of living matter and sets the foundation of the understanding of life. The following quote illustrates this hypothesis of an overarching principle which all living objects are based on, emphasizing the possibility of describing this principle of life by means of a formula:

Well, I would actually think that there is a principle that we do not yet understand, which enables life and also makes it the case that life develops from non-life. […] That there is perhaps even a connection that can be transferred in a formula that predicts that a certain increase in complexity occurs in a system, and that this then necessarily is life, right? Life necessarily arises. If it necessarily arises, you have to find a formula that describes it. We have not yet developed this formula, but I am sure that it exists. (interviewee 1)

The cited interviewee points out that there is a structuring principle in the sense of a basic physical law applying in the transition from non-living to living and enabling life understood as an abstract, overarching concept. As the development from non-living to living matter is based on a specific increase of complexity from which all forms of living matter necessarily result, the generic principle of life is considered describable by means of a mathematical formula. Following this basic concept of life, a future task for synthetic biologists is thus to discover the basic physical law or principle determining all forms of living matter in order to identify the general formula for life. This implies the expectation that research within SB will provide far-reaching new insights about the characteristics and principles of all existing forms of living matter and thereby enable the explanation of life.

Some scientific experts grouped under this conception argued that this structuring principle or connective phenomenon can be considered a consequence of the interaction between different single biological and physical features of living organisms. This implies an assumed evolution of a novel property or structure on a macro level resulting from interactions of singular elements–a process called emergence. In line with common biological as well as philosophical approaches [2, 46] these interviewees stress the fact that the whole is other than the sum of its parts, meaning that it has properties its single components do not have as they only emerge when an entity has reached a certain level of complexity and its single components interact with each other. Thus, the respondents take up the aforementioned assumed specific increase of complexity as a necessary condition for the development of living matter and bring it together with the notion of an interplay between single components of organisms. The following statement reveals this emergence of a general, hitherto undiscovered structure raising from the interactions between different properties of living objects:

If you have a system, I don’t know, of two or more elements, right, and each of them has specific features, specific characteristics, when you put those elements together, the fact that they are together and they play together, they interact together, may make it possible for the system of other properties to appear. And these new properties they are only possible because the elements are interacting between each other, this is what we call emergence. So, our properties that you cannot pre-conduct to a specific element, but only to the interaction of the elements. And there is a bunch of scientists that think life is simply an emergence property of this complex system. (interviewee 13)

As the cited statement demonstrates, within this conception life cannot be described as a list of characteristic properties shared by all forms of living matter, since it implies a yet unidentified overarching connection or structure resulting from the interactions of those features. This emerging general principle serves as the basis for all living organisms understood as “complex systems” and thus sets the foundation for an understanding of life on a general level.

#### Distinguishing dimension 1: Living versus non-living

According to the hypothesis that life follows a structuring principle enabling and determining all forms of living matter, this conceptualization hints that a basic differentiation between living and non-living can be made prospectively after identifying this overarching principle of life. Once the fundamental physical law is discovered it will be possible to draw a clear line between living and non-living objects by setting an abstract distinction of both modes of existence, which goes beyond the specific empirical processes and forms of living matter. This distinction will be based on a switch mechanism, implying a predefined starting point from which all empirical entities must be called alive due to the manifestation of the general principle of life. As scientific experts grouped under this conception assume that the principle of life is a consequence of the fact that different features of living organisms interact with each other, it can be concluded that the distinction of living and non-living is to be set precisely when a novel, overarching structure emerges resulting from the interactions of those single features. Therefore, living matter is characterized by a “high-dynamic, moved state, which maintains itself”, implying a “precarious balance or stability” (interviewee 12), arising from the interactions between the different elements. Non-living matter, on the other hand, can be understood as an “unmoved, frozen state” (interviewee 12) in which no interaction between the single features or properties takes place.

#### Distinguishing dimension 2: Natural/non-synthetic versus artificial/synthetic

With respect to the assessed relations between naturalness and artificiality within SB the respondents assumed that the generation of synthetic forms of living matter is feasible in future research activities, as the basic principles underlying synthetic and non-synthetic objects are similar. Therefore, it is not so much a question of modelling single biological properties or characteristic features, but rather a question of understanding the overarching principle connecting all different forms of living matter:

So, I can use the principles in order to construct something synthetic that has the same properties. […] If you watch a cell you often see that the collective behavior is similar to the way insects interact. So, I can describe the collective behavior of insects mathematically in the same way as the collective behavior of molecules in cells. […] They create the same structures. […] So, it is like the idea of the principles, which can actually create the same kind of organization, yes? And one can learn from this and try to make this synthetically. (interviewee 12)

As the quoted statement reveals, the construction of synthetic organisms is perceived as being possible after identifying the general principle of life, which will apply to natural as well as to synthetic forms of living organism. Again, it becomes clear that the understanding of life serves as a fundamental framework for research agendas and general objectives within SB: following this basic concept of life, a crucial aim is to discover the overarching principle determining all forms of living matter and thus representing the general formula for life in order to be able to construct novel forms of living matter from scratch. This implies the evaluation that research activities within SB are going beyond simply manipulating and imitating nature and will provide far-reaching new insights about the characteristics and principles of all existing forms of living matter, enabling the generation of synthetic organisms as a future vision of scientific results.

#### Distinguishing dimension 3: Life versus living objects

Regarding this conception of life, it can be stated that the interviewed experts differentiate between life and living objects. Both dimensions are distinguishable due to the underlying assumption that life follows a yet unknown overarching principle, which applies to all living entities existing in the empirical world. Even though this novel property or structure exists on a macro level only insofar as the living entity exists, it is distinct from the various characteristic features of the specific living object from which it emerges. According to this notion, the term “living” (“living systems” or “living matter”) addresses different specific organic forms of existence, whereas the terms “(synthetic) life” or “forms of (synthetic) life” address a generic principle, a wider and more comprehensive connection going beyond any empirical mode of existing. Hence, there is a categorical difference between single living beings and their respective characteristic features and life as an overarching concept fulfilling an organizing, structuring function.

### 3.4 Life as a relational, intuitive concept depending on subjective views

Interviewees assigned to this conception formulate life as a relational, intuitive concept, emphasizing both the individual and social construction of reality and knowledge as well as the resulting individually biased access to phenomena such as life. In line with different contemporary constructivist approaches within philosophy and social science [47, 48] they explicitly stress that the production of (scientific) knowledge is based on subjectively and socio-culturally shaped interpretations of experience as well as on relational conditions applying to perceptional processes. Hence, the perception of reality and the related production of knowledge have to be understood as processes of subjectively and socially shaped constructions by observers, which especially applies to the attempt of defining phenomena such as life and living. The following quote demonstrates this assumption particularly well:

I think that sooner or later we have to accept that the question of what life is and what non-life is substantially depends on the observer’s terminology. Actually, I would have to ask differently: if I ask ‘is an object life, living or non-living?’, then first of all I would actually have to formulate the question more carefully and ask under what circumstances a speaker can consider truthfully and determinately an object as living or non-living, and under which circumstances he cannot. And usually, we as natural scientists are convinced that the truthfulness […] of this statement depends actually only on the respective object and the statement. But I am quite sure that it depends on the object, the statement, and the speaker. And this can only be understood if one can also grasp it conceptually and intuitively. (interviewee 3)

Following these remarks, it can be stated that from an epistemological point of view an intersubjectively accepted definition of life and living cannot be provided by scientists as any definition depends on subjective ascriptions or, in other words, on constructions by the respective observer. Thus, contrary to the traditional assessment of knowledge or truthfulness in natural and life science the respondent underlines that each attempt of describing life itself as well as of distinguishing living and non-living objects is, in principle, a relational matter depending on subjective views. In line with that, he emphasizes the notion of life as an intuitive concept:

What life is and what it is not, I simply decide intuitively, as it is true for me. […] I don't think you can write an algorithm in which you insert any object and as an output you get whether it’s alive or not. I think it’s just a category mistake, it just doesn’t fit. I think it’s just a term that belongs in our intuitive world, and I leave it there, too. (interviewee 3)

As the statement reveals, the emphasis on intuitive access to phenomena such as life, living or non-living matter leads to the rejection of an assumed overarching connection that can be transferred in a formula or an algorithm, which is essential for the previously analyzed concept of life. Hence, in contrast to the understanding of life as a hitherto undiscovered general principle, interviewees grouped under this conception consider life as objectively undefinable implying that there is (and will be) no possibility to describe it by means of a scientific formula, since it is a relational, intuitive concept. Therefore, according to this basic concept of life the expectations concerning future research results within SB are lower and less comprehensive: they will not include the discovery of a physical law determining all forms of living matter and thereby enabling the explanation of the principles of life in general, but rather provide insights about characteristics of specific existing as well as novel forms of living. In doing so, the expected developments within SB might modify common understandings of living matter and life itself by evoking new subjective as well as socially shaped ascriptions.

#### Distinguishing dimension 1: Living versus non-living

As both quotes demonstrate, this conceptualization of life suggests that a scientific distinction between living and non-living matter cannot be drawn as it will constantly rely on subjective, relational, and intuitive ascriptions. The following statement of another interviewee also addresses this perspective:

So, one thing is our own perception what is living and not living, the other thing is if we try to rely on strictly defined features. […] Is a virus living or not living, I don’t really know. My personal perception is that it’s living, but this is just a logical perception like the common sense, the everyday perception. (interviewee 7)

The respondent refers to both the subjectively and socio-culturally shaped perception of what is called living and non-living, stressing that we are hardly able to establish an objective classification of both modes of existence based on generalized characteristic features met by living entities. Thus, in contrast to the second analyzed conception of life, within this conception it is not perceived as impossible to draw a line between living and non-living matter due to fluent transitions and the processuality of life (and hence due to the qualities of empirical objects themselves), but rather to the assumed fact that any distinction relies on subjective and societal ascriptions. This also implies that drawing the line between living and non-living objects can be considered a process of (social) negotiation involving the possibility of shifting boundaries between both modes of existing based on new scientific findings or the development of novel synthetic organisms.

#### Distinguishing dimension 2: Natural/non-synthetic versus artificial/synthetic

According to the relational, intuitive approach to life and living it can be stated that within this conception the generation of synthetic organisms is perceived as being possible just to a limited extent since it cannot be clearly defined whether the constructed organism is made by imitating, manipulating or constructing from scratch:

If I ask myself what life is, that is another matter than if I ask what artificial life is with an emphasis on artificial. If I say, well, when it proliferates then I simply call it life, the next question is if it is really artificial or actually only something already existing that has been recreated in a cut-down form. (interviewee 3)

Following this assumption, the extent to which novel organisms generated using SB resort to natural processes or consist of synthetically constructed components is not clearly identifiable. This is bound up with the fact that the assessment of the relations between naturalness and artificiality, again, depends on the observer’s frame of reference. Consequently, research activities within this field can be labeled as a mix of imitating nature and constructing from scratch, with both based upon the subjective judgements of the researcher.

#### Distinguishing dimension 3: Life versus living objects

Within this conception the interviewees differentiate between life and living objects on an abstract level. Following the patterns of argumentation underlying their conceptualization, it can be stated that the definition of both dimensions depends on subjective, relational and intuitive ascriptions by the respective observer(s). But while forms of living matter (called “living” or “living objects/organisms”) can principally be described by examining specific organic forms of existence within the known empirical world and by formulating a common definition based on a shared background, the phenomenon of life (called “(synthetic) life”) is understood as a universal category including all possible modes of existence and is therefore considered undefinable due to the generally limited observer’s perspective. Thus, both terms address another level of relationality: the assessment of forms of living matter relies on negotiated criteria ascribed to living objects, which are influenced by subjective interpretations as well as shared societal perceptions, norms and values and is based on the known living world or “home universe” (interviewee 3). In contrast, a definition of the phenomenon of life would have to cover an unlimited spectrum of forms of living matter that goes beyond human experience, addressing the general link between all possible modes of existing in the universe.

## 4. Discussion and conclusion

Individual understandings and implicit definitions of life affect scientific debates and research practices within SB: they have an impact on research agendas and activities related to the respective objectives and distinctive features of possible domains of investigation as well as on the definition and evaluation of forms of synthetic life [3, 49]. Our/The study most importantly shows that there are multiple and diverse underlying concepts of (synthetic-) life in the field of SB. The scientists’ concepts of life range from an understanding that underpins traditional biology and describes life as a list of properties shared by all forms of living matter [e.g. 20, 21, 43, 44, 45] to the perception that life is a non-definable, continuous process, which is in line with contemporary philosophical and scientific suggestions [23, 24, 26]. These concepts also range from a perspective that refers to common biological and philosophical concepts of emergence [2, 46] and conceptualizes life as a hitherto undiscovered general principle, to an understanding that is related to contemporary constructivist approaches within philosophy and social science [47, 48] by defining life as a relational, intuitive concept depending on subjective views. Finally, by comparing the four reconstructed conceptions of (synthetic) life and their underlying patterns of argumentation, it becomes evident that all of them, at least implicitly, refer to the same distinguishing dimensions, namely the differentiation between living and non-living, natural/non-synthetic and artificial/synthetic, and between life and living. Within each of the concepts, these distinctions serve as a basis or starting point to describe the characteristic relations and boundaries of life, although the conceptual approaches outline the respective distinctions in different ways. Thus, on a general level, the three identified distinguishing dimensions can be considered fundamental indicators to define life as a border phenomenon, which is oscillating between certain markers of differentiation, serving as dichotomous or at least distinctive poles.

The identified different basic concepts do not provide a simplified, solely mechanical understanding of life or the image of living matter as a controllable artifact, but rather imply complex conceptual approaches to life and living matter.

Nevertheless, the provided analysis has a limitation: the study is based on interviews with scientific experts working as researchers in the field of SB. Thus, the general focus of our investigation is on the scientific part of SB. However, the understanding of life of synthetic biologists working in more application-oriented, industry-driven fields aiming at developing specific applications (e.g. in medicine, biofuel production, etc.) might differ from the differentiated concepts described above or have a stronger focus on an engineering approach to living organisms. Therefore, further empirical research focusing on application-driven SB has to be done. Moreover, from a socio-ethical point of view, further research should examine the specific links to certain underlying theoretical foundations from which the reconstructed heuristic concepts derive their meaning in more detail. Within this context, an interesting further-reaching question would face the normative understandings and evaluations which underlie the different conceptions of life.

Regarding the influence of the synthetic biologists’ basic concepts of life on their scientific practice, our analysis indicates several implications on the modes of practices within SB, which will be outlined for each conceptualization in the following: within the first analyzed conception, life is defined as a stage model based on a list of biological and physical properties shared by all living matter. This understanding of life addresses the following research questions in particular: what do we have to define as the necessary and sufficient properties or features of life and living matter and how can we construct them synthetically? How simple can an organism be with regard to its properties and still be considered living? Thus, an important scientific task is to identify, examine and (re-)construct the essential features of basic biological systems, to put them together step by step and to implement them in synthetic objects through a mix of imitating nature and constructing from scratch. Accordingly, the future aim is to generate novel living organisms consisting of natural as well as synthetic components understood as building blocks. Regarding the assessment of (envisioned) scientific results, this means that novel objects constructed by means of SB might be labeled as synthetic forms of life or living matter, which would then lead to the ascription of an equal or at least similar normative status compared with non-synthetical organisms.

Scientists assigned to the second conception understand life as a non-definable, continuous process starting with the origin of life. They focus on such research questions as: where are the origins of life? How can the fluent transitions between non-living and living matter and the processuality of life be mapped and described? Therefore, within this conceptual framework, it is not a matter of identifying the essential properties of all living matter, since the differentiation between living and non-living is perceived as being not possible or meaningful. But in spite of this, the suggested research approach is similar to that of the first conception: the task is to build (partially) new objects by composing (single features within) compartments one by one. However, the objective is not to construct life itself but merely to reproduce single life-like features and processes of existing living objects synthetically. This implies that synthetic objects will still resort to natural processes and classifies the research activities within SB as imitating and manipulating nature. Regarding the evaluation of (envisioned) scientific results, this means that constructing synthetic life from scratch is not considered to be possible and, thus, partially synthetically generated objects would not be considered and treated as life or living matter.

Within the third conception, life is defined as a hitherto undiscovered general principle emerging from the interactions between the different properties of living organisms. This concept addresses the following research questions in particular: how can we describe the overarching, structuring principle determining all forms of living matter (by means of a formula)? At what point or state of interaction(s) between certain properties does the assumed specific increase of complexity from which all forms of living necessarily result happen? Hence, the task is not primarily to (re-)construct single biological and physical properties, but rather to examine how they interact with each other in order to discover the emerging basic physical law underlying all forms of living matter and thereby generate synthetic forms of living from scratch. This implies the evaluation that research activities within SB are going beyond manipulating nature and will provide far-reaching new insights about the characteristics and principles of all existing forms of living matter and life itself, enabling the generation of synthetic life as a future research aim. In line with that, future objects constructed by means of SB are considered to be living matter, implying the ascription of an equal normative status compared with non-synthetical organisms.

Researchers grouped under the fourth conception formulate life as a relational, intuitive concept depending on subjective and societally shaped views. They focus on research questions from a very fundamental perspective, asking e.g.: what are possible forms and modes of living matter (even beyond the already known)? How do relational, subjective and social perceptions shape our understanding of living matter and limit our perspective on life as a universal category? Accordingly, there are various possible domains and manners to realize the construction of synthetic forms of living matter, implying that the first general task for synthetic biologists is to distance themselves from common perceptions of life and traditional research practices. The underlying research aim is neither to provide an explanation of the principles of life by means of a formula (as within the third conception) nor to identify the properties shared by all forms of living (as within the first conception) but rather to provide insights about characteristics of specific existing as well as novel forms of living. Research activities within SB are thus labeled as a mix of imitating nature and constructing from scratch, with both based upon subjective judgements. However, developments within SB might modify familiar understandings of living matter and life itself by evoking new subjective as well as socially shaped ascriptions, which also applies with regard to the prospective normative evaluation of novel beings.

These reflections on the practical implications of scientists’ basic concepts of life show that conceptual approaches to life and living matter provide a fundamental framework for current and further scientific research within SB: they have implications for research questions, objectives, and suggested approaches as well as for the assessment of scientific results. Hence, in line with the analyzed contributions of operational definitions [3], it can be stated that the reconstructed heuristics of life have a pragmatic utility in guiding research practices within SB on a general level. As they all imply contrasting explicit or implicit assumptions on the differentiation between living and non-living matter, naturalness and artificiality with regard to the assessment of the generation of synthetic organisms, and between life and living objects the questions arises of whether and to what extent the different conceptual approaches are compatible with each other. Concerning this, we assume that there are some controversies between the different groups of scientists with respect to their very fundamental research questions and final aims, the envisioned manners and perceived general possibilities of constructing forms of synthetic life from scratch and the related evaluation of future scientific results. Nevertheless, a common current starting point for research of all groups is the (re-) construction of basic biological and physical properties as initial tools for generating novel forms of living matter. Within the present state of research in SB it can thus be expected that the distinctive understandings of life should not negatively affect cooperation between researchers with different views. However, with regard to future research activities–after reconstructing and implementing some properties synthetically–it can be expected that the controversies might lead to epistemological and practical differentiations implying different further research strategies, approaches and domains of realization. Further ethical and socio-scientific research should examine this in more detail.

Moreover, the basic issues of defining life and living matter have to be addressed within both fields of debate on SB: the rapidly developing life and bio-sciences as well as within bio-philosophy and science and technology studies. Afterwards, regulative and governance issues concerning the societal handling of (potential) novel forms of synthetic living matter could be addressed, aimed at stimulating a broad public discussion on regulatory principles of SB in particular and biotechnology in general with regard to socially acceptable forms of the modification of life and nature.
